# Association of Childhood Trauma Exposure With Adult Psychiatric Disorders and Functional Outcomes

**DOI:** 10.1001/jamanetworkopen.2018.4493

**Published:** 2018-11-09

**Authors:** William E. Copeland, Lilly Shanahan, Jennifer Hinesley, Robin F. Chan, Karolina A. Aberg, John A. Fairbank, Edwin J. C. G. van den Oord, E. Jane Costello

**Affiliations:** 1 Vermont Center for Children, Youth and Families, Department of Psychiatry, University of Vermont, Burlington; 2 The Jacobs Center for Productive Youth Development, Department of Psychology, University of Zurich, Zurich, Switzerland; 3 Department of Psychiatry, Virginia Commonwealth University, Richmond; 4 The Center for Biomarker Research and Precision Medicine, Virginia Commonwealth University, Richmond; 5 Department of Psychiatry and Behavioral Sciences, Duke University Medical Center, Durham, North Carolina

## Abstract

**Importance:**

Being exposed to trauma is a common childhood experience associated with symptoms and impairments in childhood.

**Objective:**

To assess the association between cumulative childhood trauma exposure and adult psychiatric and functional outcomes.

**Design, Setting, and Participants:**

Prospective, population-based cohort study of 1420 participants. A community representative sample of participants was assessed with structured Child and Adolescent Psychiatric Assessment interviews up to 8 times in childhood (ages 9-16 years; 6674 observations; 1993-2000) for lifetime trauma exposure as defined by the *Diagnostic and Statistical Manual of Mental Disorders*. Participants were followed up 4 times in adulthood (ages 19, 21, 25, and 30 years; 4556 observations of 1336 participants; 1999-2015) with the structured Young Adult Psychiatric Assessment Interview for psychiatric outcomes, functional outcomes, and evidence of a disrupted transition to adulthood. Analysis was completed in 2018.

**Exposure:**

Participants were assessed with the structured Child and Adolescent Psychiatric Assessment interview (parent and self-report) up to 8 times in childhood for lifetime trauma exposure (ages 9-16 years; 6674 observations; 1993-2000).

**Main Outcomes and Measures:**

Participants were assessed up to 4 times with the structured Young Adult Psychiatric Assessment interview (self-report) in adulthood (ages 19, 21, 25, and 30 years; 4556 observations of 1336 participants; 1999-2015) for psychiatric outcomes, functional outcomes, and evidence of a disrupted transition to adulthood.

**Results:**

Among the 1420 study participants, 630 (49.0%) were female and 983 (89.4%) were white. By age 16 years, 30.9% of children (n = 451) were exposed to 1 traumatic event, 22.5% (n = 289) were exposed to 2 such events, and 14.8% (n = 267) were exposed to 3 or more. Cumulative childhood trauma exposure to age 16 years was associated with higher rates of adult psychiatric disorders (odds ratio for any disorder, 1.2; 95% CI, 1.0-1.4) and poorer functional outcomes, including key outcomes that indicate a significantly disrupted transition to adulthood (eg, failure to hold a job and social isolation). Childhood trauma exposure continued to be associated with higher rates of adult psychiatric and functional outcomes after adjusting for a broad range of childhood risk factors, including psychiatric functioning and family adversities and hardships (adjusted odds ratio for any disorder, 1.3; 95% CI, 1.0-1.5).

**Conclusions and Relevance:**

Cumulative childhood trauma exposure was associated with poor adult outcomes even after accounting for many of the childhood and family factors associated with both trauma exposure and poor adult outcomes. Childhood trauma exposures are common, but often preventable, thus providing a clear target for child-focused public health efforts to ameliorate long-term morbidity.

## Key Points

**Question:**

Are adult psychiatric and functional outcomes associated with cumulative childhood trauma exposure?

**Findings:**

In this cohort study, cumulative childhood trauma was associated with higher rates of adult psychiatric disorders and poorer functional outcomes even after adjusting for a broad range of other childhood risk factors for these outcomes, including psychiatric functioning and family adversities and hardships.

**Meaning:**

Cumulative childhood trauma exposure is associated with negative outcomes in health and functioning in adulthood.

## Teaser

This cohort study examines association between cumulative childhood trauma exposure and long-term psychiatric and functional outcomes in adulthood.

## Introduction

Exposure to traumatic events (as defined by *Diagnostic and Statistical Manual of Mental Disorders* [*DSM*] criterion A for posttraumatic stress disorder)^1^ is a common experience of childhood, with more than 60% of children exposed by age 16 years and more than 30% exposed to multiple events.^2,3^ Exposure to traumatic events is associated with posttraumatic stress and other common childhood emotional and behavioral problems.^2,3,4,5^ An extensive literature links childhood trauma, particularly maltreatment, to adult psychopathology and impairment.^6,7,8,9,10^ These studies, however, have generally relied on reports of adults recalling events that occurred decades earlier.^11^ Such retrospective recalls are prone to simple forgetting or recall bias depending on the individual’s current mental health.^11,12,13^ This study uses a prospective community sample followed up repeatedly from childhood to adulthood to test whether childhood trauma has lasting effects on adult mental health and multidomain functioning.

Even if there are lasting effects of prospectively assessed trauma, these associations may be confounded by other child and family factors that commonly cluster with both trauma exposure and adult outcomes. For example, trauma exposure could merely be exacerbating premorbid emotional and behavioral symptoms that also affect adult health and functioning.^14,15,16,17^ Emotional and behavioral symptoms could be interpreted as indicators of individuals’ vulnerability and may therefore serve as a more proximal indicator of risk than trauma exposure itself. Similarly, exposure to trauma is often correlated with a broader cluster of adverse family circumstances, including socioeconomic strain, familial instability, or family dysfunction.^3,5,18,19^ The broader family context, rather than the specific exposure to trauma, may better predict long-term health and functioning.

The proposed analysis draws on a prospective, longitudinal study that assessed trauma exposure from children and their parents up to 8 times in childhood from ages 9 to 16 years. Participants were then followed up 4 times in adulthood from ages 19 to 30 years to study adult mental health and functional outcomes. Assessments prior to initial trauma exposure allow us to evaluate the potential confounding of associations between childhood trauma and adult outcomes by childhood psychiatric status and adversities. Adult outcomes included psychiatric disorders and important functional domains such as health, risky and/or criminal behavior, financial and educational status, and social functioning.

## Methods

### Participants

This report follows the Strengthening the Reporting of Observational Studies in Epidemiology (STROBE) reporting guideline for cohort studies.^20^ The Great Smoky Mountains Study is a longitudinal, representative study of children in 11 predominantly rural counties of North Carolina.^21^ Three cohorts of children, aged 9, 11, and 13 years, were recruited from a pool of some 12 000 children using a 2-stage sampling design, resulting in 1420 participants (49% female^21^). First, potential participants were randomly selected from the population using a household equal probability design. Next, participants were screened for risk of psychopathology, and participants screening high were oversampled in addition to a random sample of the rest. In addition, American Indian participants were oversampled to constitute 25% of the sample. Sampling weights were applied to adjust for differential probability of selection and to allow results to generalize to the broader population of children from which the sample was drawn. Additional details are available in the eFigure in the Supplement and previous studies.^21,22,23^

Annual assessments were completed on the 1420 children until age 16 years (6674 observations of 1420 individuals; 1993-2000) and then again at ages 19, 21, 25, and 30 years (4556 observations of 1336 participants; 1999-2015) for a total of 11 230 assessments. Interviews were completed separately by a parent figure and the participant until age 16 years, and by the participant only thereafter. Before all interviews, parent and child signed informed consent or assent forms. The study protocol and consent forms for each assessment were approved by the Duke University Medical Center institutional review board and participants received payment for their time.

### Childhood Variables

Childhood predictors of adult outcomes included the following constructs: (1) *DSM*-based traumatic events, (2) psychiatric and substance disorders, and (3) adversities and hardships. All constructs were assessed using the structured Child and Adolescent Psychiatric Assessment (CAPA).^24,25^

Cumulative childhood lifetime exposure to *DSM*-based traumatic events was assessed using the Life Events and Posttraumatic Stress sections of the CAPA. Details about the construction and psychometric properties of these sections are described elsewhere.^26^ The parent or child was queried about lifetime occurrence of each event and the timing of its occurrence. The Life Events section covered events that meet the *DSM* posttraumatic stress disorder criterion A, which stipulates that the event must involve “exposure to actual or threatened death, serious injury, or sexual violence.”^1,27^ The terms *trauma* and *traumatic events* are used to describe these events in reporting our results. eTable 1 in the Supplement includes a list of all events assessed and their frequencies in childhood. Exposure to lifetime traumatic events was aggregated into a cumulative childhood trauma exposure variable that coded 0, 1, 2, or 3 or more traumas. Traumatic events were categorized as violent trauma (including violent death of loved one, physical abuse, experiencing physical violence, war or terrorism, or captivity), sexual trauma (rape or sexual abuse), witnessing a trauma that caused or had the potential to cause death or severe injury, learning about a traumatic event occurring to a loved one, and other traumas (diagnosis with serious illness, serious unintentional injury, natural disaster, fire, or exposure to a noxious agent).

#### Childhood Psychiatric Disorders and Other Adversities and Hardships

For psychiatric symptoms, the CAPA focuses on the 3 months immediately preceding the interview to minimize recall bias. Scoring programs written in SAS statistical software (SAS Institute Inc) combine information about the date of onset, duration, and intensity of each symptom to create *DSM* diagnoses. Test-retest reliability and validity of the CAPA diagnoses are similar to other psychiatric interviews.^24,25^ Psychiatric disorders assessed included anxiety disorders, mood disorders, conduct disorder, oppositional defiant disorder, attention-deficit/hyperactivity disorder, and substance use disorders. The following categories of family hardships or childhood adversities were assessed at each observation: (1) low socioeconomic status; (2) unstable family structure (eg, single-parent family, divorce, presence of stepparent); (3) family dysfunction, including inadequate parental supervision; domestic violence; parental overinvolvement; maternal depression; marital relationship characterized by apathy, indifference, or high conflict; and high conflict between parent and child; and (4) being bullied by peers. A full description of these variables is available in a previous publication,^28^ in the eAppendix in the Supplement, and in online codebooks at http://devepi.mc.duke.edu/, which also report basic prevalence data.

### Adult Variables

All outcomes except where noted (eg, official criminal records) were assessed using the Young Adult Psychiatric Assessment (YAPA),^29^ an upward extension of the CAPA interview administered to the participants. The assessment of adult psychiatric disorders resembled that of childhood disorders, but with only self- (and not parent) reports. Disorders included any *DSM* anxiety disorder, depressive disorder, nicotine use disorder, alcohol use disorder, and cannabis use disorder. Psychosis and bipolar disorder were not included in analyses owing to very low prevalence (<1%) in the community. The participant was positive for diagnosis if criteria were met at any adult observation. Standardized scales were derived to provide a broad profile of adult functioning across 4 domains: health, risky and/or illegal behaviors, wealth (financial and/or educational), and social function. These scales were summed from dichotomous indictors in each domain (eg, college completion for wealth, smoking status for health). In some cases the indicators were positive if reported at any point in adulthood; in other cases (eg, educational attainment) the last observation was used to determine status. Standardized scores were obtained by subtracting the individual score from the group mean and dividing the resultant score by the standard deviation. A full description of all indicators used to construct these scales is available in the eAppendix in the Supplement.

### Statistical Analysis

The analytic approach must account for the 2-stage sample design. Each participant was assigned a sampling weight inversely proportional to his or her probability of selection. Next, all models used the generalized estimating equations option within SAS PROC GENMOD to derive robust variance (sandwich-type) estimates to adjust standard errors for the stratified design. Such weighted logistic (for binary outcomes such as psychiatric status), Poisson (for count variables such as number of derailments), and linear (for continuous variables such as the *z* scores for the adult function scales) regression models were used to look at differences in adult outcomes by childhood trauma status. Adjusted models account for potential confounding from childhood psychiatric problems and adversities. Consistent with common conventions, all percentages provided in the results are weighted and sample sizes are unweighted. Findings are considered statistically significant at 2-sided *P* < .05.

### Missing Data

Across all assessments, 83% of possible interviews were completed. All 1420 participants were interviewed at least once in childhood (ages 9-16 years); 1260 participants (88.7%) had 3 or more childhood observations. Of the total sample, 1336 (94.0%) were followed up at least once in adulthood at ages 19, 21, 25, or 30 years. Experiencing a childhood trauma was not associated with lower levels of participation in adulthood, suggesting no differential dropout.

## Results

### Cumulative Childhood Lifetime Trauma Exposure

Among the 1420 study participants, 630 (49.0%) were female and 983 (89.4%) were white. As previously reported,^2^ exposure to a *DSM* extreme stressor was common: 30.9% of children (451) were exposed to 1 traumatic event, 22.5% (289) were exposed to 2, and 14.8% (267) were exposed to 3 or more such events. Overall prevalence of trauma exposure did not differ by sex (although some individual traumatic events were more common in boys than girls and vice versa) or race/ethnicity. The most commonly reported events were witnessing a traumatic event (24.1%), life-threatening unintentional injuries (22.7%), and learning about an extreme stressor that occurred to a loved one (21.9%). When analyses were limited to the subset of participants with 3 or more childhood observations (1260 participants), the overall cumulative lifetime prevalence of exposure to traumatic events by age 16 years was at 70.5%, suggesting little evidence of downward bias due to attrition in childhood. Table 1 shows the prevalence and associations between cumulative lifetime childhood trauma groups and childhood psychiatric problems and other adversities and hardships. Cumulative trauma was associated with almost every type of childhood emotional and behavioral disorder and every type of childhood adversity and hardship (ie, low socioeconomic status, familial instability, family dysfunction, and being bullied by peers). eTable 2 in the Supplement shows similar associations for individual trauma categories.

**Table 1.** Prevalence of Cumulative Childhood Trauma and Unadjusted Association With Childhood Psychiatric Problems and Adversities and Hardships^a^

Psychiatric Problem, Adversity, or Hardship	Participants, No. (%)				Odds Ratio (95% CI)
	0 Exposures (n = 413)	1 Exposure (n = 451)	2 Exposures (n = 289)	≥3 Exposures (n = 267)	
Psychiatric problems					
Any	100 (14.5)	156 (26.6)	120 (33.9)	154 (46.1)	1.5 (1.3-1.8)^b^
Any anxiety disorder	27 (4.2)	56 (10.8)	47 (15.1)	64 (16.9)	1.4 (1.2-1.7)^b^
Any depressive disorder	19 (3.3)	36 (7.6)	30 (6.6)	55 (19.7)	1.7 (1.3-2.1)^b^
Attention-deficit/hyperactivity disorder	15 (2.3)	23 (3.7)	19 (3.8)	21 (4.1)	1.2 (0.9-1.5)
Oppositional defiant disorder	36 (5.7)	66 (8.8)	49 (9.7)	87 (21.4)	1.5 (1.2-1.8)^b^
Conduct disorder	36 (3.9)	48 (5.6)	46 (11.0)	73 (21.3)	1.8 (1.4-2.2)^b^
Substance use disorder	21 (4.9)	27 (5.4)	19 (6.8)	41 (11.8)	1.3 (1.0-1.7)^b^
Adversities and hardships					
Low family socioeconomic status	147 (28.1)	209 (31.4)	134 (36.5)	159 (49.4)	1.3 (1.1-1.5)^b^
Family instability	85 (16.1)	155 (25.7)	104 (29.2)	126 (47.8)	1.5 (1.3-1.8)^b^
Family dysfunction	95 (20.5)	124 (19.3)	124 (39.1)	133 (41.7)	1.5 (1.2-1.7)^b^
Bullied by peers	89 (14.7)	122 (25.5)	98 (35.5)	112 (36.8)	1.4 (1.2-1.6)^b^

^a^ All percentages are weighted and sample sizes are unweighted. Cumulative trauma exposure is treated as a continuous variable. All models adjusted for sex and race/ethnicity.

^b^ *P* < .05.

### Associations With Adult Outcomes

Of the total sample, 1336 (94.0%) were followed up at least once in adulthood at ages 19, 21, 25, or 30 years. Table 2 shows the association of cumulative lifetime trauma exposure during childhood and adult psychiatric outcomes. In models adjusted for sex and race/ethnicity only, childhood trauma status was associated with adult psychiatric status. All models were rerun adjusting for childhood psychiatric history (eg, anxiety, depressive, behavioral, and substance disorders) and childhood adversities and hardships (eg, low socioeconomic status, family instability, family dysfunction, and being bullied by peers) at the initial childhood assessment. Childhood trauma continued to predict any adult disorder and any adult anxiety disorder after adjusting for childhood covariates.

**Table 2.** Prevalence of Cumulative Childhood Trauma and Association With Adult Psychiatric Problems^a^

Psychiatric Problem	Participants, No. (%)				Adjusted Odds Ratio (95% CI)	
	0 Exposures	1 Exposure	2 Exposures	≥3 Exposures	Sex and Race/Ethnicity	Sex, Race/Ethnicity, Psychiatric Disorders, and Adversities
Any disorder	100 (37.6)	156 (49.5)	120 (47.1)	154 (54.1)	1.2 (1.0-1.4)^b^	1.3 (1.0-1.5)^b^
Any anxiety disorder	27 (12.9)	56 (18.3)	47 (14.6)	64 (26.5)	1.3 (1.0-1.5)^b^	1.3 (1.0-1.7)^b^
Any depressive disorder	19 (8.2)	36 (11.1)	30 (6.9)	55 (17.8)	1.3 (1.0-1.6)^b^	1.2 (1.0-1.6)
Any substance disorder	36 (27.1)	66 (36.8)	49 (35.7)	87 (35.9)	1.1 (1.0-1.3)	1.2 (1.0-1. 4)

^a^ All percentages are weighted and sample sizes are unweighted. Cumulative trauma exposure is treated as a continuous variable. Psychiatric diagnoses include childhood depression, anxiety, attention-deficit/hyperactivity disorder, conduct disorder, oppositional defiant disorder, and substance disorder. Childhood adversities include low socioeconomic status, familial instability, family dysfunction, and being bullied by peers.

^b^ *P* < .05.

A similar pattern of results was identified for health, risky and/or criminal behavior, financial and educational functioning, and social functioning scales (Table 3 and Figure). Childhood trauma was associated with all outcomes in models adjusted for sex and race/ethnicity and also models adjusting for childhood psychiatric problems and hardships and adversities.

**Table 3.** Associations Between Childhood Trauma Groups and Adult Functional Outcome Scales^a^

Functional Outcome	Adjusted β (95% CI)	
	Sex and Race/Ethnicity	Sex, Race/Ethnicity, Psychiatric Diagnoses, and Adversities
Health	0.20 (0.13-0.27)^b^	0.24 (0.17-0.31)^b^
Risky and/or criminal behavior	0.10 (0.03-0.16)^b^	0.12 (0.05-0.19)^b^
Financial and educational	0.19 (0.12-0.25)^b^	0.16 (0.09-0.23)^b^
Social	0.12 (0.05-0.19)^b^	0.11 (0.04-0.18)^b^

^a^ Cumulative trauma exposure is treated as a continuous variable. Psychiatric diagnoses include childhood depression, anxiety, attention-deficit/hyperactivity disorder, conduct disorder, oppositional defiant disorder, and substance disorder. Childhood adversities include low socioeconomic status, familial instability, family dysfunction, and being bullied by peers.

^b^ *P* < .05.

**Figure.**
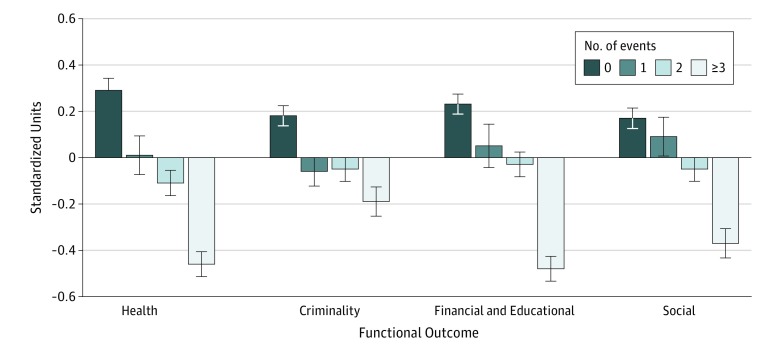
Associations Between Cumulative Childhood Trauma Exposure and Adult Outcomes Childhood trauma was associated with outcomes for health, criminality, financial and educational functioning, and social functioning. Error bars indicate standard errors.

### Moderation by Sex, Race/Ethnicity, Age at First Exposure

Interaction analyses tested whether the effects of childhood trauma differed by sex, race/ethnicity, and age at first exposure. There was little evidence that associations between childhood trauma and adult outcomes differed by either sex or race/ethnicity for adult psychiatric status or functional outcomes. With respect to age at first trauma exposure, the prospective design allowed us to split trauma exposure as having first occurred in childhood (≤12 years) or adolescence (≥13 years). Age at first trauma did not significantly moderate associations between childhood trauma and adult psychiatric history or functional outcomes except for health functioning, for which childhood trauma better predicted poor adult health than trauma in adolescence (β = 0.13; 95% CI, 0.05-0.22).

### Sensitivity Analyses

Follow-up analyses were conducted to retest findings with changes to variable definition or model specification. Many studies on childhood trauma have focused on exposure to a single trauma rather than cumulative exposure. Models for a core set of outcomes (ie, any disorder, health, and financial and educational functioning) were rerun to test how alternative definitions of trauma exposure would affect these long-term associations (Table 4). These definitions included dichotomous variables for any trauma exposure, exposure to 2 or more events, exposure to 3 or more events, or exposure to an event that was associated with childhood posttraumatic stress disorder symptoms. All alternative definitions were significantly associated with at least 1 outcome and the variable of any trauma exposure was associated with all outcomes. Next, we tested associations between specific types of trauma (ie, violent, sexual, witness trauma, learning about trauma, or other traumas) and adult outcomes (eTables 3 and 4 in the Supplement). Each trauma category was significantly associated with at least 1 adverse adult outcome; witnessing trauma was associated with multiple psychiatric and functional outcomes.

**Table 4.** Associations Between Alternative Trauma Definitions and Adult Outcomes^a^

Trauma Definition	Any Diagnosis, OR (95% CI)	β (95% CI)	
		Health	Financial and Educational
≥1 Events	1.9 (1.2 to 2.9)^b^	0.50 (0.31 to 0.68)^b^	0.32 (.14 to 0.51)^b^
≥2 Events	1.3 (0.9 to 1.9)	0.44 (0.25 to 0.64)^b^	0.18 (0.01 to 0.37)
≥3 Events	1.5 (0.9 to 2.5)	0.58 (0.34 to 0.81)^b^	0.47 (0.25 to 0.70)^b^
Any PTSD symptoms	1.4 (0.8 to 2.5)	0.08 (−0.18 to 0.33)	0.23 (0.06 to 0.52)

Abbreviations: OR, odds ratio; PTSD, posttraumatic stress disorder.

^a^ All models adjusted for sex, race/ethnicity, psychiatric history (depression, anxiety, attention-deficit/hyperactivity disorder, conduct disorder, oppositional defiant disorder, and substance disorder), and adversities (low socioeconomic status, familial instability, family dysfunction, and being bullied by peers).

^b^ *P* < .05.

Maltreatment is a distinct form of childhood trauma that is often intrafamilial and chronic and has demonstrated lasting effects on adult functioning when prospectively assessed.^30,31^ Observed associations of cumulative trauma could be driven by this particularly virulent exposure. All core outcomes continued to be significantly associated with cumulative trauma status when maltreatment was removed from the cumulative trauma variable and a maltreatment variable was added to the model (eTable 5 in the Supplement).

Finally, childhood trauma exposure may simply be associated with likelihood of trauma exposure later in life, which, in turn, may be associated with poor adult outcomes. All models were retested accounting for adult trauma exposures. Adult trauma exposure was associated with elevated risk of all adult outcomes (eTable 5 in the Supplement); nevertheless, cumulative childhood trauma also remained independently associated with adult psychiatric status and functional outcomes.

## Discussion

This study looked prospectively at associations between childhood trauma and adult outcomes. A few findings are particularly noteworthy. Childhood trauma exposure is a common experience that affects boys and girls and different racial/ethnic groups at similar rates. Such exposures are associated with an array of childhood psychiatric problems and other familial hardships and adversities. Our study suggested that childhood trauma casts a long and wide-ranging shadow, showing associations with elevated risk for adult psychiatric status, important domains of functioning (health, risky and/or criminal behavior, financial/educational functioning, and social functioning). This increased risk persisted when accounting for (1) childhood psychiatric problems, (2) other family and individual hardships and adversities, and (3) adult exposure to traumatic events.

A large body of studies has linked early adverse experiences, including traumatic events, with long-term outcomes (notably, the seminal Adverse Childhood Experiences [ACEs] studies).^32,33^ The potential for early trauma to affect behavior and functioning across the lifespan is a tenet of developmental psychopathology. Although widely accepted, support for this hypothesis has often rested on studies that assess childhood exposures retrospectively while failing to account for other childhood factors that commonly co-occur with trauma exposure. Such designs are susceptible to both recall bias and confounding.^11^ This study builds on this foundational work by adding (1) prospective, repeated assessment of childhood trauma from multiple informants, (2) measurement of a broad range of childhood factors associated with trauma exposure, (3) repeated assessments of adult functioning from age 19 to 30 years, (4) assessment of a broad range of adult functional and psychiatric outcomes, and (5) careful assessment of traumatic events experienced in adulthood. Together, these features allow the current study to address limitations in prior work and to more stringently establish the long shadow of childhood trauma.

How do these findings add to prior knowledge about potential long-term effects of early trauma? The following conclusions are noteworthy. First, rather than supporting specific effects (eg, on depression), our findings suggest that childhood trauma has broad effects on adult functioning—ranging from psychiatric status to financial and educational functioning—and these could not simply be attributed to preexisting psychiatric vulnerability or other adversities and hardships in the child’s developmental context. Previous studies had often focused on a limited number of traumas, childhood adversities, and adult outcomes, but in this study, we were able to establish these wide-ranging effects. Second, our findings provide some support for broad measurement of trauma exposure rather than focusing in on a specific trauma exposure (eg, sexual trauma).^5,34^ There may indeed be some outcome specificity to the effects of individual events,^35,36^ but the strongest and most pervasive patterns of associations are established when considering children’s total trauma history. This is consistent with recent findings on the accumulation model of trauma.^37^ Third, while children from impoverished families or violent communities are more likely to be exposed to trauma, it is still unclear which subgroups of children are at greatest risk given such exposure. Efforts to identify moderators of risk (eg, sex, race, or age) have been inconsistent and do not lend themselves to simple narratives of risk and vulnerability. The findings from this study are better suited to informing broad-based public policy efforts at reducing trauma exposure and ameliorating effects of exposure, rather than informing the development of precision medicine models to influence or predict individual response to treatment.^38^ As such, our findings support initiatives such as North Carolina’s statewide dissemination and implementation of evidence-based interventions for children with a wide range of exposures to trauma types and with varying traumatic stress reactions.^39^ Fourth, this study supported independent effects of both childhood and adult trauma exposure on adult functioning. There was limited evidence within our analyses to suggest that trauma exposure at a certain developmental period was associated with distinct subsequent risk as has been reported in multiple studies for maltreatment.^40,41^ That is, it is by no means clear that maturation—and the accumulated cognitive and emotional skills that go with it—reduces the effects of previous trauma exposure.

### Limitations

This study has many strengths, but several caveats should be kept in mind. This study is not representative of the US population. American Indians, an often understudied group, and rural areas were overrepresented in the communities from which the sample was drawn. Lifetime assessments of childhood trauma were completed annually through childhood and adolescence, but earlier experiences may have been subject to recall bias, and some traumatic events could have been forgotten—another reason to include multiple informants. Furthermore, some adverse adult effects may not be evident until later in life (eg, onset of chronic diseases). Also, although we tested for sensitive effects of first trauma exposure, such analyses rely on often unreliable reports of when participants experienced their first trauma. In addition, a broad test of timing and sensitivity effects of trauma would involve tests of individual trauma and adversity categories. Finally, additional unmeasured variables, such as genetic risk and neighborhood environmental factors, may account for aspects of observed associations.

## Conclusions

It is a myth to believe that childhood trauma is a rare experience that only affects few.^2,3^ It is similarly erroneous to believe that the primary pattern of problems in response to such trauma is characterized by posttraumatic stress symptoms. Rather, childhood trauma exposure is a normative experience, statistically speaking, that affects the majority of children at some point and subsequently has the potential to influence many aspects of functioning. This study suggests that these effects are longstanding—lasting 20 or more years—and independent of other childhood risk factors that childhood trauma tends to co-occur with. Importantly, participants’ total childhood trauma history was associated with long-term health and functioning, with each additional trauma increasing risk for adult outcomes. Together, these findings provide a clear mandate for those concerned with increasing opportunities, reducing distress, and avoiding morbidity across the lifespan. Interventions or policies that broadly target this largely preventable cluster of childhood experiences may have multifaceted effects on health and well-being that persist across the lifespan.

## Notes

Supplement. **eFigure.** Ascertainment of the Original Great Smoky Mountains Study Sample **eTable 1.** Childhood Traumatic Events and Frequencies **eAppendix.** Childhood Covariates and Adult Functional Outcomes **eTable 2.** Unadjusted Associations Between Specific Childhood Trauma Groups and Childhood Psychiatric Problems and Other Adversities/Hardships. **eTable 3.** Unadjusted Associations Between Specific Cumulative Trauma Groups and Adult Psychiatric Groups and Functional Outcomes **eTable 4.** Associations Between Specific Cumulative Trauma Groups and Adult Psychiatric Groups and Functional Outcomes Adjusted for Childhood Psychiatric Problems and Adversities/Hardships **eTable 5.** Follow-up Analyses Adjusted for Comorbid Maltreatment and Adult Trauma Exposure
