# Error in Table

**DOI:** 10.1001/jamanetworkopen.2020.7276

**Published:** 2020-05-04

In the Original Investigation titled “Association of Childhood Trauma Exposure With Adult Psychiatric Disorders and Functional Outcomes,”^1^ published November 9, 2018, the numbers of participants given in Table 2 were incorrect. This article has been corrected.^1^
