# Supplementary material for: Association of Childhood Trauma Exposure With Adult Psychiatric Disorders and Functional Outcomes
Source: JAMA Netw Open. 2018 Nov 9;1(7):e184493. doi: 10.1001/jamanetworkopen.2018.4493 (PMC6324370; doi:10.1001/jamanetworkopen.2018.4493)
Supplement: Supplement. — eFigure. Ascertainment of the Original Great Smoky Mountains Study Sample eTable 1. Childhood Traumatic Events and Frequencies eAppendix. Childhood Covariates and Adult Functional Outcomes eTable 2. Unadjusted Associations Between Specific Childhood Trauma Groups and Childhood Psychiatric Problems and Other Adversities/Hardships. eTable 3. Unadjusted Associations Between Specific Cumulative Trauma Groups and Adult Psychiatric Groups and Functional Outcomes eTable 4. Associations Between Specific Cumulative Trauma Groups and Adult Psychiatric Groups and Functional Outcomes Adjusted for Childhood Psychiatric Problems and Adversities/Hardships eTable 5. Follow-up Analyses Adjusted for Comorbid Maltreatment and Adult Trauma Exposure [file jamanetwopen-1-e184493-s001.pdf]

# Supplementary Online Content

Copeland WE, Shanahan L, Hinesley J, et al. Association of childhood trauma exposure with adult psychiatric disorders and functional outcomes. *JAMA Netw Open*. 2018;1(7):e184493. doi:10.1001/jamanetworkopen.2018.4493

**eFigure.** Ascertainment of the Original Great Smoky Mountains Study Sample
**eTable 1.** Childhood Traumatic Events and Frequencies
**eAppendix.** Childhood Covariates and Adult Functional Outcomes
**eTable 2.** Unadjusted Associations Between Specific Childhood Trauma Groups and Childhood Psychiatric Problems and Other Adversities/Hardships.
**eTable 3.** Unadjusted Associations Between Specific Cumulative Trauma Groups and Adult Psychiatric Groups and Functional Outcomes
**eTable 4.** Associations Between Specific Cumulative Trauma Groups and Adult Psychiatric Groups and Functional Outcomes Adjusted for Childhood Psychiatric Problems and Adversities/Hardships
**eTable 5.** Follow-up Analyses Adjusted for Comorbid Maltreatment and Adult Trauma Exposure

This supplementary material has been provided by the authors to give readers additional information about their work.

© 2018 Copeland WE et al. *JAMA Network Open.*

eFigure. Ascertainment of the original Great Smoky Mountains study sample

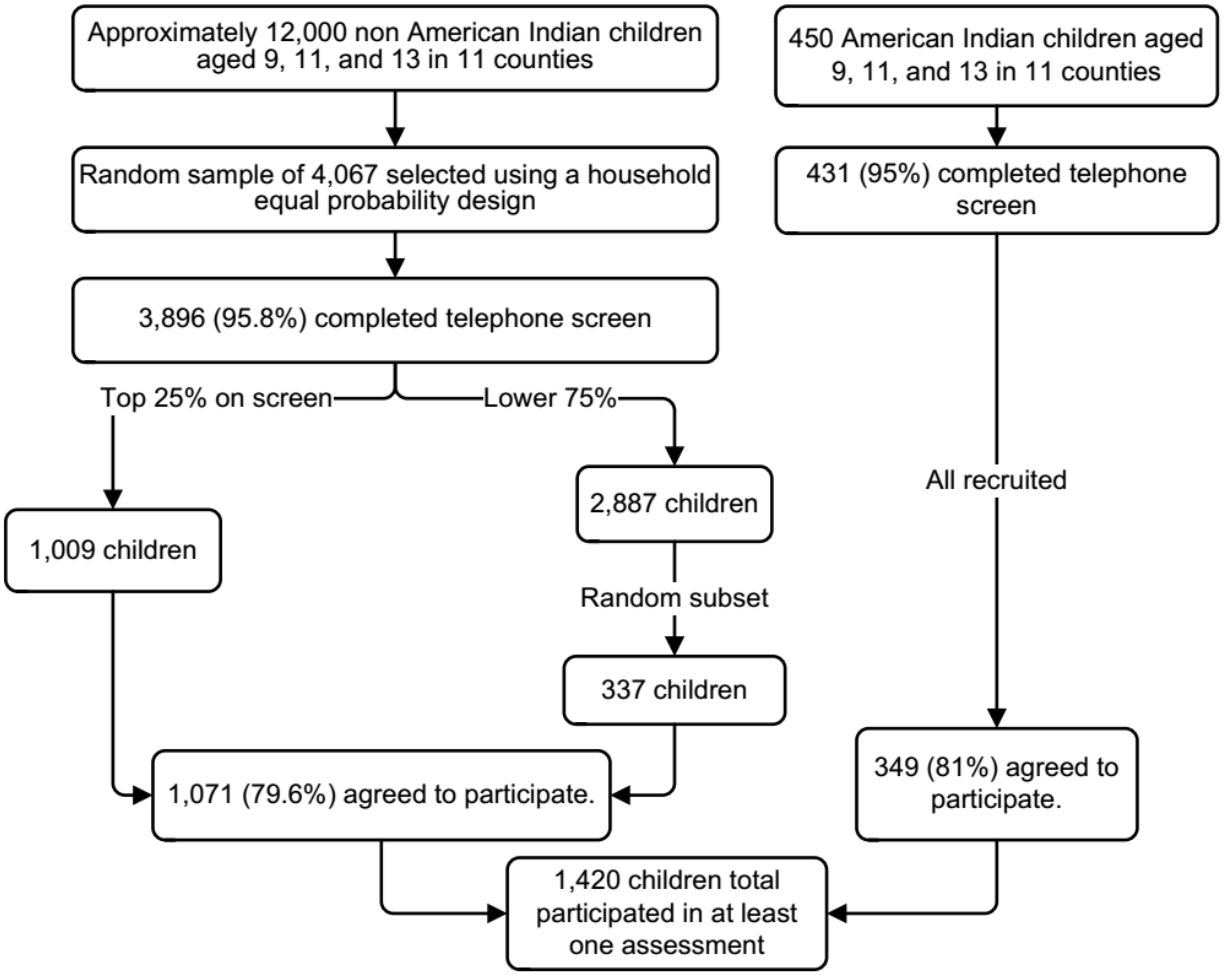


© 2018 Copeland WE et al. *JAMA Network Open.*

| eTable 1. Childhood traumatic events and frequencies | |
|---|---|
| | **%** |
| **Violence** | **24.7** |
| Violent Death of loved one | 2.4 |
| Violent Death of sib/peer | 14.5 |
| War, terrorism | 0.1 |
| Cause of death or severe harm | .6 |
| Victim of physical violence | 3.1 |
| Physical abuse by relative | 7.2 |
| Captivity | .9 |
| **Sexual trauma** | **11.0** |
| Sexual abuse | 10.9 |
| Rape | 1.2 |
| Coercion | 4.3 |
| **Other Injury or Trauma** | **32.8** |
| Diagnosis of Physical illness | 11.0 |
| Serious accident | 11.6 |
| Natural disaster | 11.1 |
| Fire | 5.9 |
| Exposure to noxious agent | 3.3 |
| **Witness to life event** | **23.7** |
| **Learned about life event** | **21.4** |
| **Any Trauma** | **67.8** |
| **Number of Trauma** | |
| 1 event | 30.8 |
| 2 or more event | 37.0 |

All percentages are weighted.

© 2018 Copeland WE et al. *JAMA Network Open.*

**eAppendix. Childhood covariates and adult functional outcomes**
Definition for childhood covariates:

Four types of *family adversities/hardships were assessed:* low socioeconomic status (SES), unstable family structure, family dysfunction, and maltreatment. Low SES was positive if the child's family met 2 or more of the following conditions: below the US federal poverty line based upon family size and income, parental high school education only, or low parental occupational prestige[35]. Unstable family structure was positive if child's family met 2 or more of the following conditions: single parent structure, step-parent in household, divorce, parental separation, or change in parent structure. Family dysfunction was positive if child's family met 5 or more of the following conditions: inadequate parental supervision of child's free time, over-involvement of the parent into the child's activities in an age-inappropriate manner, physical violence between parents, top 20% in terms of frequency of parental arguments, marital relationship characterized by absence of affection, apathy, or indifference, child is upset by or actively involved in arguments between parents, mother scores in elevated range on depression questionnaire, top 20% in terms of frequency of arguments between parent and child, and most parental activities are source of tension or worry for the child. Peer victimization was positive if child or parent reported that the child had been a regular target of bullying or harassment by peers. Codebooks for all items available at http://devepi.duhs.duke.edu/codebooks.html.

© 2018 Copeland WE et al. *JAMA Network Open.*

Adult functional Outcomes:

Health. Participants reported being *diagnosed with a serious illness* or being in a *serious accident* at any point during young adulthood. Having a *sexually transmitted disease* was coded if the participant reported testing positive for herpes, genital warts, chlamydia, or HIV. At each assessment, weight and height measurements were used to derive body mass index. *Obesity* was defined by CDC definitions up to age 20 and greater than or equal to 30 $kg/m^2$ thereafter. Participants were assessed for a *DSM-IV psychiatric diagnosis* including the following: any DSM-IV anxiety disorder, any depressive disorder, and antisocial personality disorder. *Regular smoking* was defined as smoking > 1 cigarette per day for 3 months. A physical health problems survey adapted from Form HIS-1A (1998), US Department of Commerce for the U.S. Public Health Service was administered at all interviews. This assessed self-reported *perceived poor health*, *high illness contagion risk*, and *slow illness recovery*.

Risky/illegal behaviors. Official felony charges were harvested from NC administrative Offices of the Courts records. Self-report was used to assess recent *police contact*, often *lying* to others, *frequent physical fighting*, and *breaking into another home/business/property*. Frequent *drunkenness* was coded if subject reported drinking to excess at least once weekly for 3 months. Subjects reported recent use of *marijuana* or other *illegal substances*. Finally, subjects reported having one-time sexual encounters with strangers (*hooking up with strangers*).

Wealth: Financial/educational accomplishments**.** *Being impoverished* was coded based upon thresholds issued by the Census Bureau based on income and family size [36]. *High school*

*dropout* and *completion of any college education* were coded based upon the subject's educational status at the last adult assessment. The following job problems were assessed at each assessment: *being dismissed or fired* from a job and *quitting a job* without financial preparations. Finally, other financial problems assessed included: *failing to honor debts or financial obligations* and being *a poor manager of one's finances*.

Social relationships**.** *Marital, parenthood,* and *divorce* status were determined through self-report at the last adult assessment. The quality of the participant's relationship with their parents, spouse/significant other, and friends was assessed at each assessment including arguments and violence. Variables were included to indicate any *violence in a primary relationship*, *poor relationship with one's parents*, *no best friend or confidante*, and *problems making or keeping friends*.

© 2018 Copeland WE et al. *JAMA Network Open.*

| eTable 2.Unadjusted associations between specific childhood trauma groups and childhood psychiatric problems and other adversities/hardships. | | | | | | | | | | |
|---|---|---|---|---|---|---|---|---|---|---|
| | Violent trauma | | Sexual Trauma | | Other Trauma | | Witness Trauma | | Learned about Trauma | |
| | OR | CI | OR | CI | OR | CI | OR | CI | OR | CI |
| **Psychiatric problems** | | | | | | | | | | |
| Any | **2.2** | **1.5-3.4** | **2.2** | **1.3-3.8** | 1.2 | 0.8-1.8 | 2.4 | **1.6-3.7** | **2.4** | **1.6-3.7** |
| Any anxiety dx. | **1.9** | **1.1-3.2** | 1.5 | 0.8-2.7 | 1.6 | 0.9-2.7 | **2.3** | **1.4-4.0** | **1.9** | **1.1-3.4** |
| Any depressive dx. | **2.1** | **1.1-3.9** | **2.6** | **1.3-5.1** | **2.2** | **1.2-4.0** | **2.7** | **1.4-5.0** | **1.9** | **1.0-3.6** |
| ADHD | **2.6** | **1.1-5.7** | 1.6 | 0.7-3.5 | 0.8 | 0.4-1.6 | 0.8 | 0.4-1.5 | **2.3** | **1.0-5.3** |
| ODD | **2.2** | **1.3-3.5** | **3.2** | **1.7-6.1** | 1.0 | 0.6-1.7 | **2.6** | **1.6-4.3** | **1.7** | 1.0-2.8 |
| CD | **2.5** | **1.4-4.3** | **4.4** | **2.1-9.2** | 1.5 | 0.9-2.6 | **3.3** | **1.9-5.7** | 2.3 | 1.3-4.2 |
| Substance | 1.4 | 0.7-3.0 | 1.2 | 0.5-3.1 | 0.8 | 0.4-1.6 | **2.4** | **1.2-4.9** | **3.1** | **1.5-6.4** |
| | | | | | | | | | | |
| **Adversities/Hard-ships** | | | | | | | | | | |
| Low family SES | **1.7** | **1.1-2.5** | **2.6** | **1.5-4.5** | 0.9 | 0.6-1.4 | **2.0** | **1.3-3.0** | **1.8** | **1.2-2.8** |
| Family instability | **3.2** | **2.1-4.8** | 1.6 | 0.9-2.7 | 1.1 | 0.7-1.6 | **2.6** | **1.7-4.0** | **1.9** | **1.2-2.9** |
| Family dysfunction | **2.3** | **1.6-3.5** | **1.7** | **1.0-2.9** | 1.2 | 0.8-1.8 | **3.2** | **2.1-4.9** | 1.5 | 0.9-2.2 |
| Peer victimization | **1.7** | **1.1-2.6** | **2.8** | **1.6-4.9** | 1.2 | 0.8-1.9 | **2.3** | **1.5-3.5** | 1.2 | 0.7-1.8 |

All percentages are weighted and ns are unweighted. Cumulative trauma exposure is treated as a continuous variable. All models adjusted for sex and race/ethnicity. Bolded values are significant at $p < 0.05$. OR= odds ratio; CI = 95% confidence intervals; Dx=disorder.

© 2018 Copeland WE et al. *JAMA Network Open.*

| eTable 3. Unadjusted associations between specific cumulative trauma groups and adult psychiatric groups and functional outcomes. | | | | | | | | | | |
|---|---|---|---|---|---|---|---|---|---|---|
| | Violent trauma | | Sexual Trauma | | Other Trauma | | Witness Trauma | | Learned about Trauma | |
| | OR | CI | OR | CI | OR | CI | OR | CI | OR | CI |
| **Psychiatric problems** | | | | | | | | | | |
| Any disorder | 1.1 | 0.7-1.6 | 1.2 | 0.7-2.2 | **1.5** | **1.0-2.1** | **1.8** | **1.2-2.7** | 1.3 | 0.8-2.0 |
| Any anxiety dx. | 1.5 | 0.9-2.5 | 1.8 | 0.9-3.4 | 1.3 | 0.8-2.2 | **1.8** | **1.1-3.0** | 1.0 | 0.5-1.7 |
| Any depressive dx. | 1.4 | 0.8-2.4 | **2.2** | **1.0-4.5** | 1.4 | 0.8-2.5 | **2.0** | **1.1-3.6** | 1.2 | 0.6-2.3 |
| Any substance dx. | 0.9 | 0.6-1.4 | 0.9 | 0.5-1.7 | **1.4** | **1.0-2.2** | 1.5 | 0.9-2.3 | 1.4 | 0.9-2.2 |
| | | | | | | | | | | |
| **Functional outcomes** | | | | | | | | | | |
| Health | **.38** | **.15-.60** | .23 | -.09-.56 | **.40** | **.19-.60** | **.21** | **.01-.42** | .19 | -.02-.42 |
| Risky/criminal | .06 | -.14-.26 | -.11 | -.35-.13 | **.26** | **.08-.45** | **.25** | **.06-.43** | **.19** | **-.02-.41** |
| Financial | **.44** | **.23-.65** | **.40** | **.15-.65** | .13 | -.05-.32 | **.38** | **.18-.58** | .19 | -.02-.41 |
| Social | **.36** | **.14-.57** | .21 | -.07-.49 | .01 | -.17-.20 | **.38** | **.18-.58** | .09 | -.10-.28 |

All percentages are weighted and ns are unweighted. Cumulative trauma exposure is treated as a continuous variable. All models adjusted for sex and race/ethnicity. Bolded values are significant at p <0.05. OR= odds ratio; CI = 95% confidence intervals; Dx=disorder.

© 2018 Copeland WE et al. *JAMA Network Open.*

| eTable 4. Associations between specific cumulative trauma groups and adult psychiatric groups and functional outcomes adjusted for childhood psychiatric problems and adversities/hardships. | | | | | | | | | | |
|---|---|---|---|---|---|---|---|---|---|---|
| | Violent trauma | | Sexual Trauma | | Other Trauma | | Witness Trauma | | Learned about Trauma | |
| | OR | CI | OR | CI | OR | CI | OR | CI | OR | CI |
| **Psychiatric problems** | | | | | | | | | | |
| Any disorder | 1.1 | 0.7-1.6 | 1.2 | 0.7-2.2 | **1.5** | **1.0-2.1** | **1.8** | **1.2-2.7** | 1.3 | 0.8-2.0 |
| Any anxiety dx. | 1.5 | 0.9-2.5 | 1.8 | 0.9-3.4 | 1.3 | 0.8-2.2 | **1.8** | **1.1-3.0** | 1.0 | 0.5-1.7 |
| Any depressive dx. | 1.4 | 0.8-2.4 | **2.2** | **1.0-4.5** | 1.4 | 0.8-2.5 | **2.0** | **1.1-3.6** | 1.2 | 0.6-2.3 |
| Any substance dx. | 0.9 | 0.6-1.4 | 0.9 | 0.5-1.7 | **1.4** | **1.0-2.2** | 1.5 | 0.9-2.3 | 1.5 | 0.9-2.3 |
| | | | | | | | | | | |
| **Functional outcomes** | | | | | | | | | | |
| Health | **.39** | **.18-.59** | .23 | -.09-.56 | **.45** | **.26-.65** | .18 | -.02-.37 | .21 | -.01-.42 |
| Risky/criminal | .05 | -.14-.24 | -.08 | -.32-.15 | **.31** | **.13-.51** | **.24** | **.05-.44** | **.19** | **.01-.40** |
| Financial | **.34** | **.14-.54** | **.31** | **.08-.54** | .17 | -.01-.34 | **.24** | **.04-.43** | .14 | -.07-.35 |
| Social | **.29** | **.09-.49** | .20 | -.06-.46 | .05 | -.12-.22 | **.27** | **.08-.46** | .07 | -.10-.24 |

All percentages are weighted and ns are unweighted. Cumulative trauma exposure is treated as a continuous variable. All models adjusted for sex, race/ethnicity, psychiatric history (depression, anxiety, ADHD, conduct, oppositional defiant and substance disorders), and adversities (low SES, familial instability, family dysfunction, and peer victimization). Bolded values are significant at $p < 0.05$. OR= odds ratio; CI = 95% confidence intervals; Dx=disorder.

© 2018 Copeland WE et al. *JAMA Network Open.*

| eTable 5. Follow-up analyses adjusted for comorbid maltreatment and adult trauma exposure | | | | | | | | | |
|---|---|---|---|---|---|---|---|---|---|
| | **Any dx.** | | | **Health** | | | **Financial/Educational** | | |
| | OR | CI | P | MR | CI | p | MR | CI | p |
| | | | | | | | | | |
| Controlled for: | | | | | | | | | |
| Maltreatment | **1.3** | **1.0-1.5** | **0.03** | **.27** | **.18-.37** | **<0.001** | **.16** | **.07-.24** | **0.002** |
| Recent trauma | 1.2 | 1.0-1.5 | 0.06 | **.21** | **.14-.29** | **<0.001** | **.14** | **.07-.21** | **<0.001** |

All models adjusted for sex, race/ethnicity, psychiatric history (depression, anxiety, ADHD, conduct, oppositional defiant and substance disorders), and adversities (low SES, familial instability, family dysfunction, and peer victimization). Bolded values are significant at p <0.05.

© 2018 Copeland WE et al. *JAMA Network Open.*
